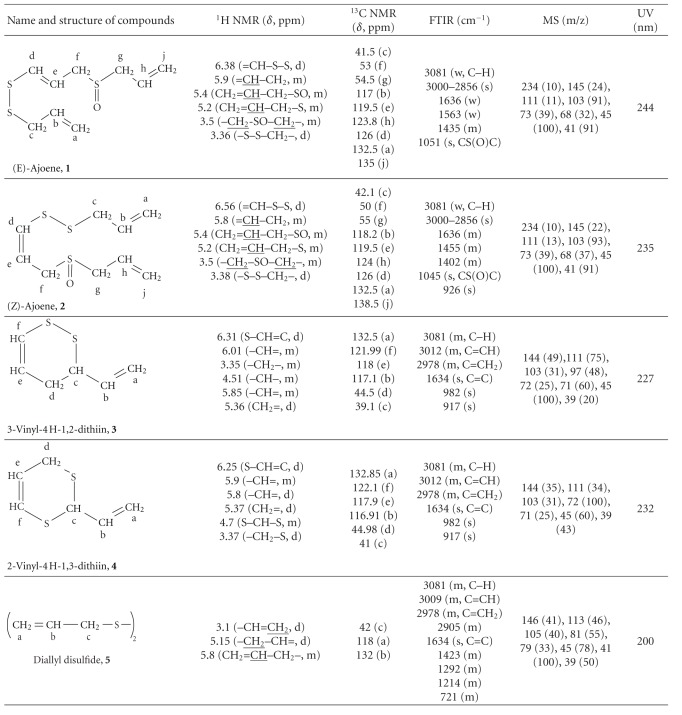# Transformation of Synthetic Allicin: The Influence of Ultrasound, Microwaves, Different Solvents and Temperatures, and the Products Isolation

**DOI:** 10.1100/2012/561823

**Published:** 2012-05-02

**Authors:** Dušica Ilić, Vesna Nikolić, Mihajlo Stanković, Ljubiša Nikolić, Ljiljana Stanojević, Ivana Mladenović-Ranisavljević, Andrija Šmelcerović

**Affiliations:** ^1^Faculty of Technology, University of Niš, Leskovac, Serbia; ^2^Department of Pharmacy, Faculty of Medicine, University of Niš, Niš, Serbia

## Abstract

The transformation of the synthesized allicin, using conventional method, the influence of ultrasound and microwaves, in different organic solvents (acetonitrile, acetone, methanol, and chloroform), at various temperatures (room temperature, 45°C, and 55°C) was investigated. Allicin degradation kinetic was monitored by HPLC. Allicin transformation under the effect of microwaves is faster than transformations performed under the influence of ultrasound or by conventional method. Increase of the temperature accelerates allicin transformation. Pharmacologically active compounds of (E)-ajoene, (Z)-ajoene, 3-vinyl-4H-1,2-dithiin, 2-vinyl-4H-1,3-dithiin, and diallyl disulfide were isolated from the mixture of transformation products of allicin under the influence of microwaves in methanol at 55°C, which is according to kinetic parameters (highest values of the order of reaction and the lowest activation energy) the optimal method.

## 1. Introduction

Allicin (3-prop-2-enylsulfinylsulfanylprop-1-ene) is a thioester of sulfenic acid, or allylthiosulfinate. It is an oily, light yellow liquid that has a distinctively pungent smell [1]. Allicin is the most important pharmacologically active substance of the fresh aqueous extract of garlic [2–4] which exhibits antimicrobial [5–10], antiviral [11], antioxidant [7], anticancer [12], antihypertensive [13], and hypolipidemic activity [13]. Isolation of allicin from garlic is a very complex and difficult process because of its instability. The synthesis of allicin is based on the oxidation of allyl disulfide with hydrogen peroxide in acidic media [14–17], the oxidation of allyl disulfide with 3-chloroperoxybenzoic acid in chloroform [18], and the oxidation of diallyl disulfide with magnesium peroxyhydrate in the presence of ammonium butyl sulphate [19]. In our previous paper on the synthesis of allicin [20] we proposed radical mechanism of allyl disulfide oxidation. Due to the significant instability of allicin, synthesis procedures are usually performed at temperatures from 0°C to 25°C.

**Scheme 1:**
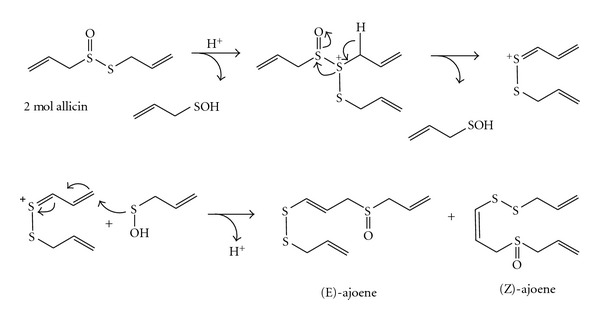
Mechanism of allicin transformation to ajoenes [21].

**Scheme 2:**
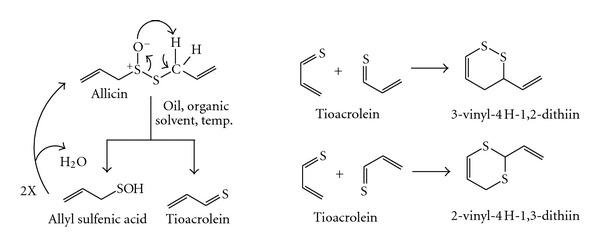
Transformation of allicin in oil and organic nonpolar solvents [21].

Allicin was more stable in 20% alcohol than in water, but surprisingly unstable in vegetable oil, with half-life activity of 0.8 hours [22]. In order to increase the stability of allicin, we obtained the inclusion complexes with *β*-cyclodextrin [8] and carbamide [9], where its pharmacological activity was still preserved. There are data about the transformation of allicin in the polar [21, 23] and nonpolar [1, 3, 4] media, where active products with higher stability than allicin were obtained. In hexane or soybean oil, allicin forms ajoenes and vinyldithiins [4]. Block and collaborators have proposed degradation mechanisms of allicin to the above-mentioned products (Schemes 1 and 2) [21].

In our previous paper [7] we examined the stability of synthesized allicin by FTIR method, at room temperature. Herein, we present the influence of various factors (ultrasound, microwaves, different solvents, and temperatures) on the kinetics of transformation of allicin. To the best of our knowledge, this is the first report on the influence of ultrasound and microwaves on the allicin transformation. For the optimal transformation process, according to kinetic parameters, from the mixture of transformation products by preparative HPLC method, predominant compounds were isolated. Their structures were determined by spectroscopic methods (UV, FTIR, NMR, and MS).

## 2. Experimental

### 2.1. Synthesis of Allicin

Allicin was synthesized according to our previous procedure [20]. Synthesis is based on the oxidation of allyl disulfide with the acidic hydrogen peroxide. Allicin was extracted from the crude reaction mixture using multiple liquid-liquid extraction with diethyl ether.

### 2.2. Transformation of Allicin in the Presence of Solvent at Room Temperature

Transformation of allicin (1 g) in organic solvents (9 cm^3^ of acetonitrile, acetone, methanol, or chloroform) was obtained using conventional method, the influence of ultrasound and microwaves.

Conventional transformation of allicin has been performed for seven days at room temperature and by heating in a water bath with reflux at 45°C and 55°C.

Transformation under the influence of ultrasound was performed in the ultrasonic bath (Sonic, Nis, Serbia; total nominal power: 3 × 50 W; dimensions of the bath: 30 × 15 × 20 cm) at the frequency of 40 kHz, with reflux at 45°C and 55°C.

Transformation under the effect of microwaves was performed in a “Discover” focus microwave reactor (CEM Corporation, Matthews, NC, USA), at a frequency of 2.45 GHz, with power of 150 W, at 45°C and 55°C.

### 2.3. Determination of Allicin Content

The content of allicin was analyzed using HPLC-UV method. Chromatographic analysis was performed using Agilent 1100 system equipped with an Agilent 1200 autosampler. Separations were performed on Zorbax Eclipse XDB-C18 (4.6 × 250 mm, 5 *μ*m) column (Agilent, Santa Clara, USA). The mobile phase consisted of acetonitrile and water, 80 : 20, v/v. The flow rate was 1 mL/min and the injection volume was 20 *μ*L. All separations were performed at 25°C. DAD detection was performed using a surveyor Agilent photodiode array detector at 205 nm.

External calibration was performed in the range of 12.5 *μ*g/mL to 250 *μ*g/mL. Within the range of concentrations injected, the detector response (peak area) was linear. Correlation coefficient for the calibration curve was 0.998.

### 2.4. Isolation and Structural Determination of Transformation Products

Isolation of transformation products was performed using preparative HPLC chromatography, on the Agilent Technologies 1200 Series device. Separations were performed on a Zorbax XDB-C-18 (6.2 × 150 mm, 5 *μ*m) column (Agilent, Santa Clara, USA). The mobile phase is consisted of acetonitrile and water, 80 : 20, v/v. The flow rate was 4 mL/min and the injection volume was 70 *μ*L. Seventy separations were performed at 25°C. DAD detection was performed using a surveyor Agilent photodiode array detector at 205 nm. For each isolated component an UV spectrum has been recorded.

FTIR spectra were recorded at Bomem MB-100 spectrophotometer (Hartmann & Braun, Baptiste, Canada) using the KBr technique in the range of 4000–400 cm^−1^.

^1^H-NMR and ^13^C-NMR measurements were performed by a Bruker AC 250E (Bruker, Germany) spectrometer at the operating frequencies of 250 MHz and 62.5 MHz, respectively, using deuterated chloroform as solvent.

MS analysis of allicin transformation products was performed using a LCQ Fleet Ion Trap LC-MS^n^ system (Thermo Scientific, San Jose, USA). Chromatographic conditions were identical with those of the determination of allicin content. Mass spectra were obtained in positive ionization mode using an extractor voltage of 4.5 kV. 

## 3. Results and Discussion

**Figure 1:**
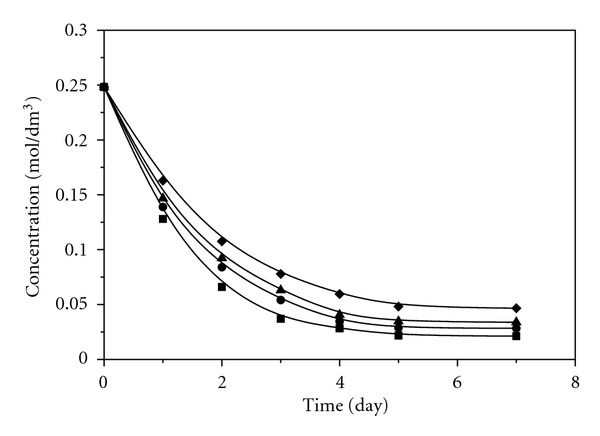
Change of allicin concentrations in acetonitrile, acetone, chloroform, and methanol at room temperature (■ Acetonitrile; ● Acetone; ▲ Methanol 55°C; **♦ **Chloroform).

**Figure 2:**
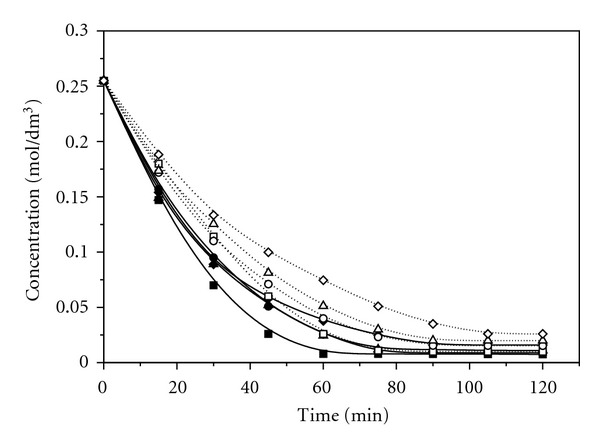
Change of the allicin concentration using conventional heating at 45°C and 55°C (■ Acetonitrile 55°C; □ Acetonitrile 45°C; ● Acetone 55°C; ∘ Acetone 45°C; ▲ Methanol 55°C; *▵* Methanol 45°C; **♦ **Chloroform 55°C; *◊* Chloroform 45°C).

Figure 1 shows changes in concentrations of allicin during the time, at room temperature, in acetonitrile, acetone, methanol, and chloroform. The most significant change of the allicin concentration occurs in the first five days in all solvents used. The lowest concentration of allicin remaining was detected in acetonitrile, while the highest concentration was detected in chloroform. Transformation of allicin in the above-mentioned solvents, using conventional method at higher temperatures (45°C and 55°C), was significantly faster than the same process at room temperature (Figure 2). The most significant change of the allicin concentration in acetonitrile was achieved in about 75 minutes, while in chloroform it was achieved in about 120 minutes. Also, after the transformation at temperature of 55°C the lower concentrations of allicin remaining were detected in all solvents used compared to the results obtained at room temperature.

**Figure 3:**
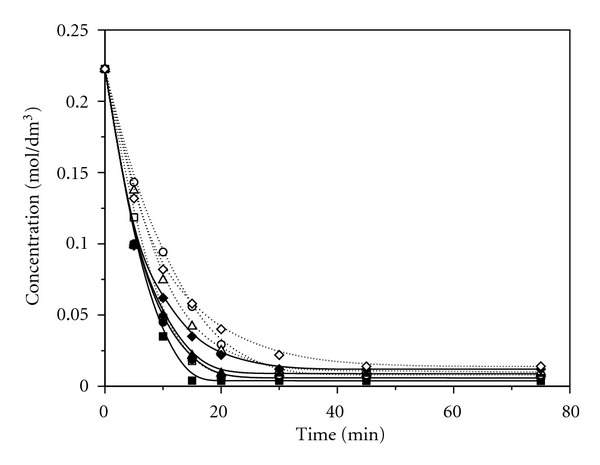
Change of the allicin concentration under the influence of ultrasound at 45°C and 55°C (■ Acetonitrile 55°C; □ Acetonitrile 45°C; ● Acetone 55°C; ∘ Acetone 45°C; ▲ Methanol 55°C; *▵* Methanol 45°C; **♦ **Chloroform 55°C; *◊* Chloroform 5°C).

Transformation of allicin under the influence of ultrasound (Figure 3) occurs three to four times faster compared to the conventional process of transformation in all solvents used. In these transformations, as well as in conventional ones, the lowest concentration of the allicin remaining was found in acetonitrile.

**Figure 4:**
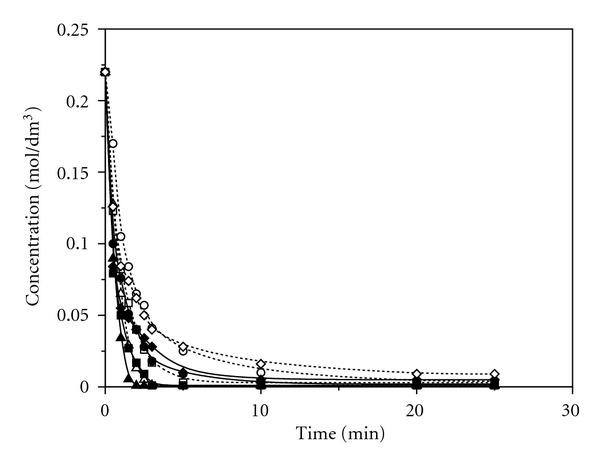
Change of allicin concentration under the influence of microwaves at 45°C and 55°C (■ Acetonitrile 55°C; □ Acetonitrile 45°C; ● Acetone 55°C; ∘ Acetone 45°C; ▲ Methanol 55°C; *▵* Methanol 45°C; **♦ **Chloroform 55°C; *◊* Chloroform 45°C).

Microwaves have a bigger impact on the transformation rate, compared to the ultrasound and the conventional process, for the same temperatures and solvents used (Figure 4). The fastest transformation including the complete transformation of allicin was achieved in methanol at 55°C in about 2 minutes.

In order to be able to compare the abilities of different solvents to generate heat from microwave irradiation, their capabilities to absorb microwave energy and to convert the absorbed energy into heat, must be taken into account. Those factors may be considered using the loss angle, **δ**, which is usually expressed in the form of its tangent

(1) $$\tan\delta \;\;=\;\frac{\varepsilon^{\prime \prime }}{\varepsilon^{\prime }}.$$

The dielectric constant, *ε*′, represents the ability of dielectric material to store electrical potential energy under the influence of an electric field. The loss factor, *ε*′′, quantifies the efficiency with which the absorbed energy is converted into heat [24]. The transformation of allicin occurs fastest in methanol which can be explained by the fact that this solvent has a much higher value of *δ* (0.659) compared to the other three solvents, whose *δ* values range from 0.054 to 0.091 [24].

Kinetic parameters of allicin transformations were determined by the reaction rate equation

(2) $$-\frac{dC_{A}}{dt}=k\cdot C_{A}^{n}.$$

Logarithm of this equation is given in (3). By using this equation, kinetic parameters of the reaction of allicin transformation, chemical reaction rate constant (*k*), and order of reaction (*n*) can be determined

(3) $$\ln\left(-\frac{dC_{A}}{dt}\right)=\ln\left(k\right)+n\cdot \ln\left(C_{A}\right).$$

**Figure 5:**
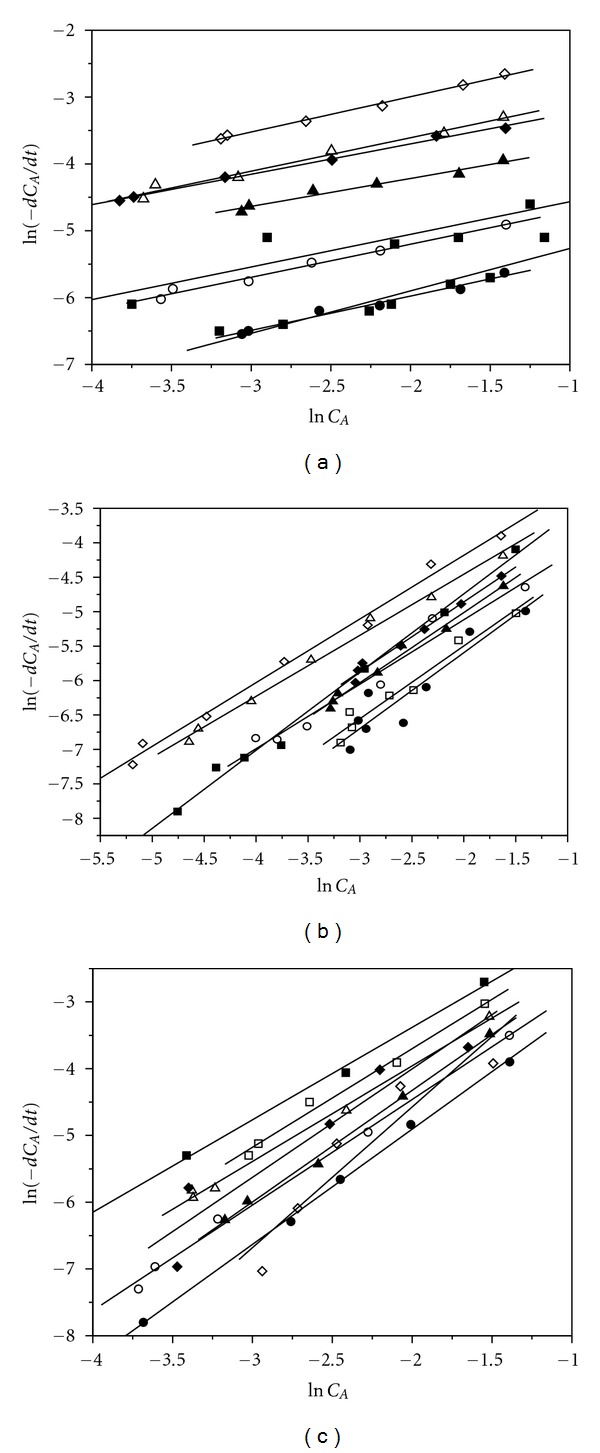
Dependence ln⁡⁡(−*dC*_*A*_/*dt*) of ln⁡⁡(*C*_*A*_) for the allicin transformation by conventional method (a), under the influence of ultrasound (b) and microwaves (c). (▪ Acetonitrile 55°C; □ Acetonitrile 45°C; ● Acetone 55°C; ∘ Acetone 45°C; ▲ Methanol 55°C; *▵* Methanol 45°C; **♦ **Chloroform 55°C; *◊* Chloroform 45°C).

For all the transformations, dependence ln(−*dC*_*A*_/*dt*) of ln(*C*_*A*_) is shown in Figure 5. Activation energy (Ea) was determined from Arrhenius's equation

(4) $$k=A\cdot e^{-\text{Ea}/RT}.$$

**Figure 6:**
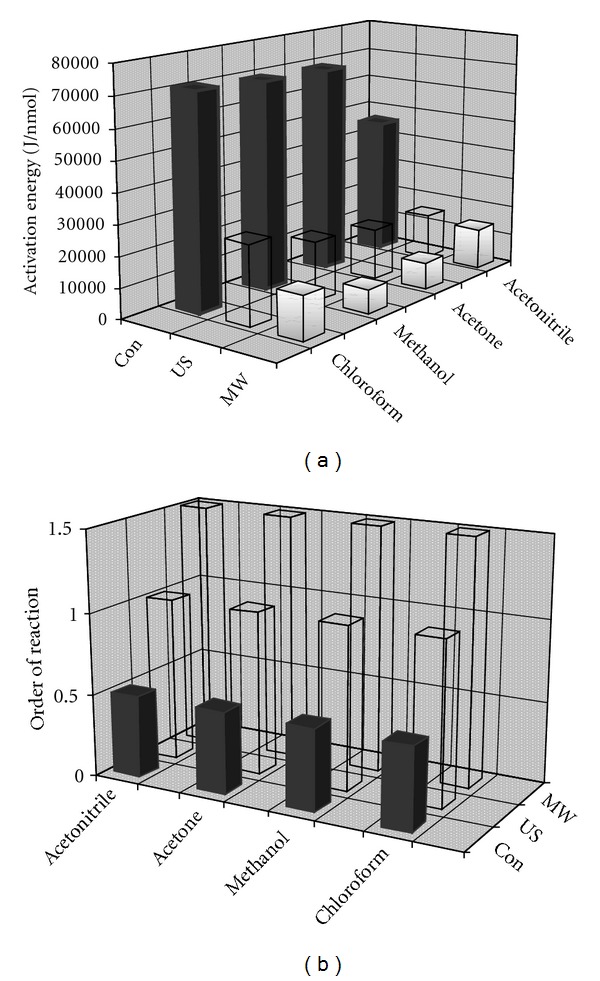
Values of Ea (a) and *n* (b) for the allicin transformation by conventional method (Con), under the influence of ultrasound (US) and microwaves (MW).

The changes of Ea and *n* for the applied solvents and techniques of allicin transformation are shown in Figure 6. Under the effect of microwaves in all solvents used, the highest value of *n* (1.5) is achieved. Under the influence of ultrasound *n* equals 1, while in the conventional process it equals 0.5. The highest values of Ea are required for the conventional method of allicin transformation, while the lowest Ea values are required for the process under the effect of microwaves. According to Ea values, the most suitable allicin transformation process is performed under the influence of microwaves in methanol at 55°C (Ea = 7902 J/mol). Also, this process occurs within the shortest transformation time (about 2 minutes). The highest Ea value is required for the conventional procedure in chloroform at 45°C (Ea = 70645 J/mol) and this process requires the longest time of transformation. Therefore, these conditions are least suitable for allicin transformation.

**Figure 7:**
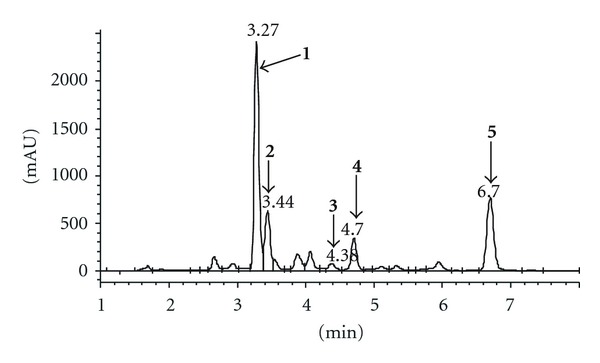
HPLC chromatogram of the mixture of allicin transformation products obtained under the effect of microwaves in methanol at 55°C ((E)-ajoene (**1**); (Z)-ajoene (**2**); 3-vinyl-4H-1,2-dithiin (**3**); 2-vinyl-4H-1,3-dithiin (**4**); dialildisulfid (**5**)).

The most common components (see Figure 7) from the mixture of transformation products of allicin under the influence of microwaves in methanol at 55°C were isolated using preparative HPLC chromatography. Their structures were determined by UV, FTIR, NMR, and MS methods and spectroscopic data are given in Table 1. (E)-Ajoene ((E)-1-(prop-2-enyldisulfanyl)-3-prop-2-enylsulfinylprop-1-ene; (**1**), (Z)-ajoene ((Z)-1-(prop-2-enyldisulfanyl)-3-prop-2-enylsulfinylprop-1-ene; (**2**), 3-vinyl-4H-1,2-dithiin (3-ethenyl-3,4-dihydrodithiine; (**3**), 2-vinyl-4H-1,3-dithiin (2-ethenyl-4H-1,3-dithiine; (**4**), and diallyl disulfide (3-(prop-2-enyldisulfanyl)prop-1-ene; (**5**) were isolated. **1 **and **2** exhibit antimicrobial [25, 26] and anticancer effect [27, 28]. **3** and **4** participate in the inhibition of thrombocyte aggregation, cyclooxygenase and 5-lipoxygenase inhibition, and regulation of systolic and diastolic blood pressure [29]. **5** inhibits 1,2-dimethylhydrazine-induced colon and liver cancer in rodents [30]. Presence of these compounds in the transformation mixture is in accordance with the previous findings about the ways of allicin transformation [20].

Due to the complexity of the mixture of transformation products other compounds could not be isolated.

## 4. Conclusion

The transformation rate of allicin depends on the applied techniques of transformation, temperature, and solvents nature. Use of microwaves and increased temperatures accelerate the allicin transformation. Kinetic parameters, *n* and Ea, depend on the transformation techniques used. The highest *n* and the lowest Ea values were achieved under the influence of microwaves in methanol at temperature of 55°C.

## Figures and Tables

**Table 1:** Spectral data for the components isolated from the mixture of allicin transformation products obtained under the effect of microwaves in methanol at 55°C.